# Predictive value of LncRNA on coronary restenosis after percutaneous coronary intervention in patients with coronary heart disease: A protocol for systematic review and meta-analysis

**DOI:** 10.1097/MD.0000000000024114

**Published:** 2021-01-08

**Authors:** Shengxiang Liu, Guokang Yang, Yupeng Huang, Cheng Zhang, Hongyan Jin

**Affiliations:** Department of Cardiology, Hanyang hospital of Wuhan University of Science and Technology, Hanyang, Wuhan,China.

**Keywords:** biomarker, coronary heart disease, coronary restenosis, diagnosis, long-stranded non-coding RNA, meta-analysis, percutaneous coronary intervention

## Abstract

**Background.:**

Evidence shows that long-stranded non-coding RNA (LncRNA) can predict coronary artery restenosis in patients suffering from coronary heart disease after percutaneous coronary intervention, suggesting that LncRNA may become a promising biomarker for the diagnosis of coronary artery restenosis after percutaneous coronary intervention. However, its accuracy has not been systematically evaluated. Therefore, it is necessary to perform meta-analysis to certify the diagnostic value of LncRNA on coronary artery restenosis after percutaneous coronary intervention.

**Methods.:**

PubMed, EMBASE, Cochrane Library, and Web of Science were searched for relevant studies to explore the potential diagnostic values of LncRNA on coronary artery restenosis after percutaneous coronary intervention from inception to December 2020. Data were extracted by two experienced researchers independently. The risk of bias about the meta-analysis was confirmed by the Quality Assessment of Diagnostic Accuracy Studies-2 (QUADAS-2). Data was synthesized and heterogeneity was investigated as well. All of the above statistical analysis was carried out with Stata 14.0.

**Results.:**

This study proved the pooled diagnostic performance of LncRNA on coronary artery restenosis after percutaneous coronary intervention.

**Conclusion.:**

This study clarified confusions about the specificity and sensitivity of LncRNA on coronary artery restenosis after percutaneous coronary intervention, thus further guiding their promotion and application.

**Ethics and dissemination.:**

Ethical approval is not required for this study. The systematic review will be published in a peer-reviewed journal, presented at conferences, and shared on social media platforms. This review would be disseminated in a peer-reviewed journal or conference presentations.

**OSF registration number:**

DOI 10.17605/OSF.IO/4QT2P.

## 1 Introduction

As a common cardiovascular disease, coronary heart disease poses a serious threat to human health.^[1]^ Percutaneous coronary intervention is an effective technique for clinical treatment of coronary heart disease, and can greatly improve the survival rate of patients with coronary heart disease.^[2,3]^ However, the problem of in-stent restenosis seriously influences the effect of percutaneous coronary intervention, which still perplexes clinical workers.^[4]^ More than 98% non-protein coding RNA and nucleotides in human genome are long-stranded non-coding RNA (LncRNA) with more than 200 nucleotides that can regulate myocardial hypertrophy, myocardial fibrosis and cardiomyocyte apoptosis.^[5–7]^

Previous studies have proved the abnormal expression of LncRNA in patients with coronary restenosis after percutaneous coronary intervention, indicating the potential diagnostic value of LncRNA.^[8–11]^ Nonetheless, the inadequate sample size, inconsistent subjects or diverse detection techniques lead to considerable discrepancy among those studies and inconsistent results. In this study, we evaluated current literatures that focus on the association between LncRNA and coronary artery restenosis after percutaneous coronary intervention by meta-analysis.

## 2 Methods

### 2.1 Study registration

The protocol of the systematic review has been registered on Open Science Framework (registration number: DOI 10.17605/OSF.IO/4QT2P). It was reported following the guideline of Preferred Reporting Items for Systematic Reviews and MetaAnalysis Protocol statement.^[12]^

### 2.2 Inclusion criteria for study selection

#### 2.2.1 Type of studies

To explore the diagnostic value of LncRNA on the diagnosis of coronary artery restenosis after percutaneous coronary intervention.

#### 2.2.2 Type of participants

All patients with coronary heart disease after percutaneous coronary intervention were included in this review, regardless gender, age, and population.

#### 2.2.3 Type of index test

Index test: LncRNA was applied to detect patients with coronary restenosis after percutaneous coronary intervention. However, we excluded case reports, reviews, cell, or animal studies.

#### 2.2.4 Outcome measurements

Outcomes include Pooled sensitivity (SEN), specificity (SPE), positive likelihood ratio (PLR), negative likelihood ratio (NLR), diagnostic odds ratio (DOR), area under the curve (AUC), and their 95% confidence intervals (CIs).

### 2.3 Data sources and search strategy

This study conducted a literature search in PubMed, EMBASE, Cochrane Library, and Web of Science. We made a final search on December 4, 2020. The search strategy of Pubmed is displayed in Table 1. We adopted similar retrieval strategies for other electronic databases.

**Table 1:** Search Strategy (PubMed).

Number	Search terms
1	Coronary Disease[MeSH]
2	Coronary Heart Disease[Title/Abstract]
3	Coronary Diseases[Title/Abstract]
4	Coronary Heart Diseases[Title/Abstract]
5	Disease, Coronary[Title/Abstract]
6	Disease, Coronary Heart[Title/Abstract]
7	Diseases, Coronary[Title/Abstract]
8	Diseases, Coronary Heart[Title/Abstract]
9	Heart Disease, Coronary[Title/Abstract]
10	Heart Diseases, Coronary[Title/Abstract]
11	or/1–10
12	Percutaneous Coronary Intervention[MeSH]
13	Percutaneous Coronary Revascularization[Title/Abstract]
14	Coronary Intervention, Percutaneous[Title/Abstract]
15	Coronary Interventions, Percutaneous[Title/Abstract]
16	Coronary Revascularization, Percutaneous[Title/Abstract]
17	Coronary Revascularizations, Percutaneous[Title/Abstract]
18	Intervention, Percutaneous Coronary[Title/Abstract]
19	Interventions, Percutaneous Coronary[Title/Abstract]
20	Percutaneous Coronary Interventions[Title/Abstract]
21	Percutaneous Coronary Revascularizations[Title/Abstract]
22	Revascularization, Percutaneous Coronary[Title/Abstract]
23	Revascularizations, Percutaneous Coronary[Title/Abstract]
24	or/12–23
25	Coronary Restenosis[MeSH]
26	Coronary Restenoses[Title/Abstract]
27	Restenoses, Coronary[Title/Abstract]
28	Restenosis, Coronary[Title/Abstract]
29	or/25–28
30	Long-stranded non-coding RNA
31	LncRNA
32	or/30–31
33	diagnos^∗^
34	sensitivity
35	specificity
36	ROC curve
37	or/33–36
38	11 and 24 and 29 and 32 and 37

### 2.4 Data collection and analysis

#### 2.4.1 Study selection

**Figure 1:**
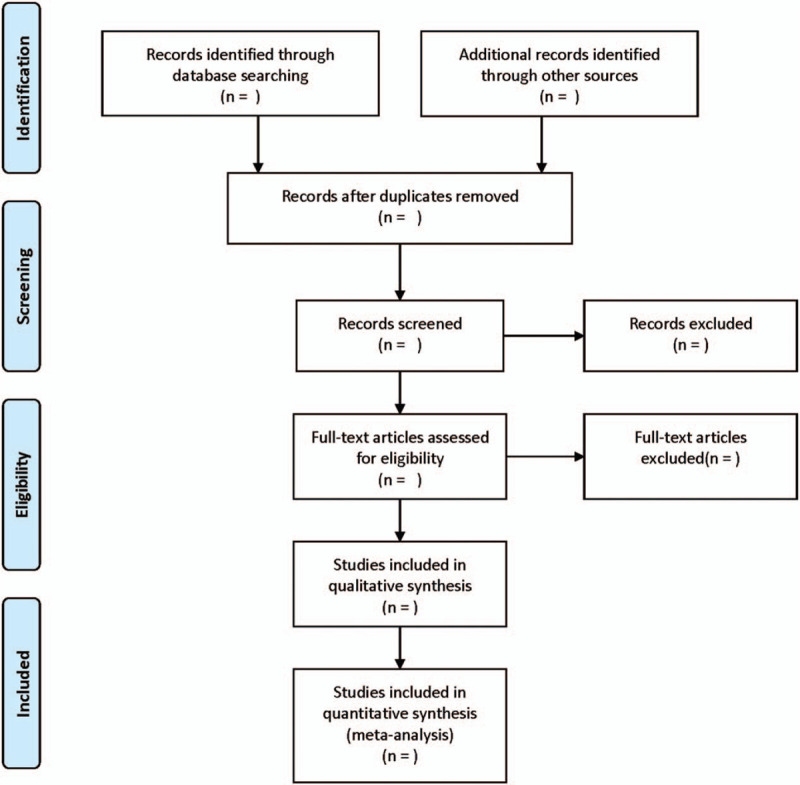
Flow diagram showing literature filtration process.

Two reviewers will screen the titles and abstracts independently. Afterwards, according to the inclusion criteria, the full text of potential studies will be retrieved for further selection. Any disagreements will be resolved by a third researcher. The screening flow chart of this study is demonstrated in Figure 1.

#### 2.4.2 Data extraction

Two reviewers independently extracted the data with a standardized form and the data were confirmed by a third researcher. In case of missing or unclear information, we contacted the authors to clarify them. The data extraction form includes following items: first author, publication year, regions, sample size, sample types, control group, studied LncRNA, LncRNA detection methods, and data needed for diagnostic meta-analysis.

### 2.5 Quality assessment

The methodological quality of the included studies was assessed following Quality Assessment of Diagnostic Accuracy Studies-2 (QUADAS-2) criteria. The tool consists of four key domains: patient selection, index test, reference standard, and flow and timing, aiming to evaluate the risk of the bias and concerns about clinic applicability of all included publications. Two reviewers independently and blindly checked the studies. Any discrepancies between the two reviewers were resolved by consensus.

### 2.6 Statistical analysis

All of the above statistical analysis was performed with Stata 14.0 (Stata Corp, College Station, TX). We calculated the pooled SEN, SPE, PLR, NLR, DOR, and their 95% CI. In addition, the pooled diagnostic value of LncRNA through the summary receiver operating characteristic curve (SROC) and AUC was tested. The threshold effects were tested by using spearman correlation coefficient. The calculation of heterogeneity was caused by the non-threshold effect of Cochrane-*Q* and *I*^*2*^ values, and a fixed effect model (without obvious inhomogeneity) or a random effects model (with significant heterogeneity) was employed to merge the data. The statistical test level was α = 0.05.

### 2.7 Subgroup analysis

In order to further investigate potential heterogeneity, subgroup analyses were carried out based on ethnicity, LncRNA type, source of LncRNA, and sample size.

### 2.8 Sensitivity analysis

We conducted a sensitivity analysis to evaluate the robustness of our analysis.

### 2.9 Reporting bias

The Deeks symmetry test was carried out to detect whether there is a publication bias in the included studies.

### 2.10 Ethics and dissemination

The content of this article does not involve moral approval or ethical review and will be presented in print or at relevant conferences.

## 3 Discussion

LncRNA can regulate gene expression at the transcriptional and post-transcriptional level, participate in the growth and development of the body, and maintain at a relatively stable level, and its abnormal expression is abnormal in malignant tumors, cardiovascular, nervous system and other diseases. It has been reported that it can be regarded as a diagnostic marker and therapeutic target.^[13–16]^ LncRNA may participate in the process of atherosclerosis by regulating the proliferation and apoptosis of vascular smooth muscle cells. Meanwhile, it may be one of the mechanisms of genetic susceptibility to coronary heart disease, and can provide a basis for the early screening of coronary heart disease.^[17,18]^ Although many studies indicated abnormal LncRNA expression in patients with coronary restenosis after percutaneous coronary intervention, a systematic review and meta-analysis will be warranted to compile and synthesize the available data to address some of the questions.

This is the first meta-analysis to comprehensively search and summarize the evidence of the effect of LncRNA on the diagnosis of coronary restenosis after percutaneous coronary intervention in patients with coronary heart disease. The results of this review could provide clinical evidence and represent a possibility and future direction of coronary restenosis after percutaneous coronary intervention in patients with coronary heart disease diagnosis.

## Author contributions

**Data curation:** Shengxiang Liu, Guokang Yang.

**Methodology:** Cheng Zhang.

**Project administration:** Hongyan Jin.

**Software:** Guokang Yang, Yupeng Huang.

**Validation:** Yupeng Huang.

**Visualization:** Shengxiang Liu.

**Writing – original draft:** Hongyan Jin, Shengxiang Liu.

**Writing – review & editing:** Hongyan Jin, Shengxiang Liu.

## References

[1] Inaguma D Koide S Takahashi K Relationship between history of coronary heart disease at dialysis initiation and onset of events associated with heart disease: a propensity-matched analysis of a prospective cohort study. BMC Nephrol 2017;18:79. 28245790 10.1186/s12882-017-0495-8 PMC5331727

[2] Jiang H Zhang H Yang Y Associations of myeloperoxidase, interleukin-17A and heparin-binding EGF-like growth factor levels with in-stent restenosis after percutaneous coronary intervention: a single-centre case-control study in China. BMJ Open 2020;10:e039405. 10.1136/bmjopen-2020-039405 PMC7651712 33158827

[3] Kurtul A Elcik D Procalcitonin is an independent predictor for coronary atherosclerotic burden in patients with stable coronary artery disease. Int J Cardiol 2017;236:61–4. 28256322 10.1016/j.ijcard.2017.02.061

[4] Hee L Terluk A Thomas L Late clinical outcomes for SeQuent please paclitaxel-coated balloons in PCI of instent restenosis and de novo lesions: A single-center, real world registry. Catheter Cardiovasc Interv 2017;89:375–82. 27113534 10.1002/ccd.26546

[5] Bink DI Lozano-Vidal N Boon RA Long non-coding RNA in vascular disease and aging. Noncoding RNA 2019;5:26. 10.3390/ncrna5010026 PMC6468806 30893946

[6] Molkentin JD Bugg D Ghearing N Fibroblast-specific genetic manipulation of p38 mitogen-activated protein kinase in vivo reveals its central regulatory role in fibrosis. Circulation 2017;136:549–61. 28356446 10.1161/CIRCULATIONAHA.116.026238 PMC5548661

[7] van Almen GC Verhesen W van Leeuwen RE MicroRNA-18 and microRNA-19 regulate CTGF and TSP-1 expression in age-related heart failure. Aging Cell 2011;10:769–79. 21501375 10.1111/j.1474-9726.2011.00714.x PMC3193380

[8] Wang H Gong H Liu Y Relationship between lncRNA-Ang362 and prognosis of patients with coronary heart disease after percutaneous coronary intervention. Biosci Rep 2020;40:1–0. 10.1042/BSR20201524 PMC7383831 32686826

[9] Zhang Z Gao W Long QQ Increased plasma levels of lncRNA H19 and LIPCAR are associated with increased risk of coronary artery disease in a Chinese population. Sci Rep 2017;7:7491. 28790415 10.1038/s41598-017-07611-z PMC5548926

[10] Yin LP Zhang HM Tan H Relationship between serum LncRNA GAS5 level and post-PCI in-stent restenosis in elderly patients with coronary heart disease. Chin J Evid Based Cardiovasc Med 2020;12:1176–84.

[11] Zhang H Xu Y Guo MK The predictive value of serum LncRNAMIR155HG in coronary artery restenosis in patients with coronary heart disease after PCI. Chin J Evid Based Cardiovasc Med 2020;12:984–7.

[12] Shamseer L Moher D Clarke M Preferred reporting items for systematic review and meta-analysis protocols (PRISMA-P) 2015: elaboration and explanation. BMJ 2015;350:g7647. 25555855 10.1136/bmj.g7647

[13] Niland CN Merry CR Khalil AM Emerging roles for long non-coding RNAs in cancer and neurological disorders. Front Genet 2012;3:25. 22375145 10.3389/fgene.2012.00025 PMC3286759

[14] Hewitt KJ Sanalkumar R Johnson KD Epigenetic and genetic mechanisms in red cell biology. Curr Opin Hematol 2014;21:155–64. 24722192 10.1097/MOH.0000000000000034 PMC6061918

[15] Yu LM Xu Y Epigenetic regulation in cardiac fibrosis. World J Cardiol 2015;7:784–91. 26635926 10.4330/wjc.v7.i11.784 PMC4660473

[16] Valente S Liu Y Schnekenburger M Selective non-nucleoside inhibitors of human DNA methyltransferases active in cancer including in cancer stem cells. J Med Chem 2014;57:701–13. 24387159 10.1021/jm4012627 PMC3983372

[17] Challen GA Sun D Jeong M Dnmt3a is essential for hematopoietic stem cell differentiation. Nat Genet 2011;44:23–31. 22138693 10.1038/ng.1009 PMC3637952

[18] Watson CJ Collier P Tea I Hypoxia-induced epigenetic modifications are associated with cardiac tissue fibrosis and the development of a myofibroblast-like phenotype. Hum Mol Genet 2014;23:2176–88. 24301681 10.1093/hmg/ddt614

